# RAGE targeted strategies for Alzheimer’s amyloid β peptide induced blood brain barrier dysfunctions

**DOI:** 10.1186/1750-1326-8-S1-P5

**Published:** 2013-09-13

**Authors:** Sholpan Askarova, Andrey Tsoy, James C-M Lee

**Affiliations:** 1 Center for Life Sciences, Nazarbayev University, Astana, Kazakhstan; 2 Department of Biological Engineering, University of Missouri, Columbia, MO, USA

## Background

Blood-brain barrier (BBB) dysfunctions have been implicated in the development and progression of Alzheimer’s disease. Cerebral endothelial cells (CECs) and astrocytes are the main cell components of the BBB. Although amyloid-β oligomers have been reported to mediate oxidative damage to the CECs and astrocytes and trigger the downstream MAPK/ERK pathway, the cell surface binding site for Aβ and exact sequence of these events have yet to be elucidated. In this study, the receptor for advanced glycation endproducts (RAGE) was postulated to function as a signal transducing cell surface receptor for Aβ to induce ROS generation from NADPH oxidase and trigger downstream pathways for the phosphorylation of extracellular-signal-regulated kinases (ERK1/2) and cytosolic phosphorilase A_2_ (cPLA_2_).

## Materials and methods

Immortalized CECs (bEnd3) and primary rat astrocytes were applied in this study. To confirm Aβ-RAGE binding, we examined the quantitative immunofluorescence microscopy (QIM) of cellular surface RAGE for primary astrocytes and bEnd3 cells pretreated with oligomeric Aβ1-42 at +4°C (conditions in which the internalization of surface receptors is suppressed) and stained with antiRAGE primary antibody (Ab_RAGE_). To quantify the ROS production, we applied fluorescence microscopy of dihydroethidium (DHE) in the cells. Confocal immunofluorescence microscopy of double immunofluorescent labeled gp91-phox and p47-phox subunits was performed to characterize NADPH oxidase complex assembly. Western blot analysis was applied to characterize phosphorylation of cPLA2α.

## Results

We report that Aβ_42_ competed with the anti-RAGE antibody (Ab_RAGE_) to bind to RAGE on the surfaces of CECs and primary astrocytes. In addition, Ab_RAGE_ abrogate Aβ_42_-induced ROS production and the co-localization between the cytosolic (p47-phox) and membrane (gp91-phox) subunits of NADPH oxidase in both cell types. Ab_RAGE_, as well as NADPH oxidase inhibitor and ROS scavenger suppressed Aβ_42_-induced ERK1/2 and cPLA_2_ phosphorylation in CECs. At the same time, only Ab_RAGE_, but not NADPH oxidase inhibitor or ROS scavenger, inhibited ERK1/2 and cPLA2α phosphorylation in primary astrocytes.

## Conclusions

This study demonstrated that Aβ_42_ binding to RAGE on the CEC and astrocyte surfaces is required for NADPH oxidase complex assembling, resulting in subsequent ROS generation and phosphorylation of ERK1/2 and cPLA_2_ and suggested the presence of two different RAGE-dependent downstream pathways in CECs and astrocytes. Therefore, understanding precise molecular mechanisms underlying Aβ mediated oxidative damage may provide new insights into the development of preventive and treatment strategies for AD.

